# Breast cancer risk is associated with the HULC rs7763881, MTMR3 rs12537 polymorphisms, and serum levels of HULC and MTMR3 in Egyptian patients

**DOI:** 10.1007/s11033-023-08897-1

**Published:** 2023-11-01

**Authors:** Mona Elhelaly, Olfat G. Shaker, Ghada Ayeldeen, Alyaa R. Elsergany, Nora Mostafa

**Affiliations:** 1 Medical Biochemistry and Molecular Biology Department, Faculty of Medicine, Mansoura University, Mansoura, Egypt; 2 Medical Biochemistry and Molecular Biology Department, Faculty of Medicine, Cairo University, Cairo, Egypt; 3 Internal Medicine Department, Medical Oncology Unit, Oncology Center, Faculty of Medicine, Mansoura University, Mansoura, Egypt

**Keywords:** Breast cancer, Polymorphism, LncRNAs, MTMR3, HULC

## Abstract

**Background:**

Highly upregulated in liver cancer (HULC) is one of the LncRNAs that was documented to enhance cancer progression, and its downregulation is associated with cell cycle arrest and apoptosis. Myotubularin-related protein 3 (MTMR3) is required for autophagy, and many studies consider MTMR3 to be a negative regulator of autophagy processes. However, nothing is understood about how they regulate breast cancer.

**Material and methods:**

This case–control study included 245 patients (Group A: 85 early BC Group B: 40 metastatic BC cases, Group C: 40 fibroadenoma cases; and Group D: 80 age matched healthy control subjects. TaqMan Real-time PCR was used to analyse rs7158663 and rs12537. MTMR3 and HULC gene expression levels were measured using RT-PCR.

**Result:**

Breast cancer patients exhibited elevated serum MTMR3 and HULC compared to fibroadenomas and control cases. The MTMR3 rs12537 “T/T” genotype was highly expressed in cases of breast cancer (early and metastatic) compared to controls (risk genotype). On the other hand, the HULC rs7158663 genotypes were not statistically associated with breast cancer. However, when compared to the control, the C/C genotype of the HULC gene is higher in the case.MTMR3 gene expression was higher in the T/T genotype compared to both the C/C and C/T genotypes, while HULC gene expression was lower in the A/C genotype compared to both the A/A and C/C genotypes. Positive correlation between MTMR3 and HULC. MTMR3 and ALT, as well as HULC and alkaline phosphatase, both showed a statistically significant positive correlation.

**Conclusion:**

Our findings reveal that MTMR3 and HULC serum expression and their SNPs (HULC rs7763881, MTMR3 rs12537) are associated with a higher risk for the development of breast cancer in the Egyptian population.

**Supplementary Information:**

The online version contains supplementary material available at 10.1007/s11033-023-08897-1.

## Introduction

Breast cancer is the most common cancer in women, with an incidence rate of 10.4 percent of all cancers. In terms of cancer deaths, breast cancer is thought to account for 15% of all cancer-related deaths [1]. In Egypt, it constitutes 33% of female cancer cases, and more than 22,000 new cases are diagnosed each year [2].

LncRNAs are non-coding RNA consists of more than 200 nucleotides, and it has been documented to have an important role in the regulation of many genetic events that affect carcinogenesis, such as epigenetic events, gene expression regulation, including translational and post-transcriptional regulation, and many biological processes, including cancer progression, tumor suppressor genes, and oncogenes. In addition to their role in many biological processes, LncRNAs are abnormally expressed in many cancers, which may increase the potential to use them as a noninvasive diagnostic biomarker [3, 4].

Highly upregulated in liver cancer (HULC) is one of the LncRNAs that is highly expressed in liver cancer cells and plays a vital role in many cellular processes, including cell proliferation and apoptosis. It is reported that HULC enhances cancer progression and that its downregulation is associated with activation of p53 and increased expression of p21, finally leading to cell cycle arrest and apoptosis [5, 6].

According to numerous studies, HULC can stimulate the progression of many different cancers, including ovarian carcinoma, prostate cancer, and pancreatic cancer. However, it is yet unknown how HULC regulates breast cancer [7, 8].

The myotubularin (MTM) family member myotubularin-related protein 3 (MTMR3) was initially discovered as an inositol lipid 3 phosphatase that can hydrolyze phosphatidylinositol 3-phosphate (PI3P) [9, 10].

Because PI3P is required for autophagy, many studies consider MTMR3 to be a negative regulator of autophagy processes [11, 12].

It has been shown that MTMR3 has a role in the development of tumors, including oral and colon cancer, and that autophagy is an important intracellular mechanism that acts as a tumour inhibitor to increase cell survival by impacting tumour growth and metastasis. But nothing is understood about how it functions in breast cancer [13].

### Aim of the study

We looked into the blood expression levels of HULC and MTMR3 genes, their correlation with clinicopathological data, and their potential as non-invasive biomarkers in fibroadenoma and BC patients. We also investigated the association of their SNPs (HULC rs7763881 and MTMR3 rs12537) with the susceptibility to breast cancer (BC) and fibroadenoma.

## Material and methods

This is a case–control study that was conducted at the Medical Biochemistry Department, Faculty of Medicine, Mansoura University, Egypt, Medical Biochemistry and Molecular Biology Department, Faculty of Medicine, Cairo University, Egypt; and the Medical Oncology Unit at the Oncology Center at Mansoura University, Egypt.

All participants in this study signed informed consent after being informed about all steps of the study, including the publication step. The study follows the ethical guidelines of the 1975 Declaration of Helsinki. This study was approved by an ethics committee at the Faculty of Medicine, Mansoura University.

The Inclusion criteria were: (1) patients diagnosed with breast cancer without receiving chemotherapy or radiotherapy, diagnosis and cancer staging was done by Mansoura Medical Oncology Center (2) patients with complete clinical data.

The Exclusion criteria were: (1) All patient with other types of cancers (2) Patients who stared chemotherapy, radiotherapy or surgical removal (3) Patients with incomplete clinical data.

This study involved 245 Egyptian females from the Medical Oncology Department, Mansoura University (from May 2020 to May 2022). To confirm our diagnosis, mammography and surgical biopsies were used. Cases were divided into four groups:

- *Group A*: 85 non-metastatic BC cases (All participants were newly diagnosed females, before receiving chemotherapy, antihormonal, or radiotherapy treatment. Tumor size, tumour type, and TNM staging were involved in our study)
- *Group B*: 40 metastatic BC cases before receiving any type of breast cancer treatment (tumor grade, clinical size, metastasis, tumour type, and lymph node involvement were included in our clinical report).
- *Group C*: 40 fibroadenoma cases (age, parity, menstrual history, and family history were included in our clinical report).
- *Group D*: 80 age-matched healthy control subjects (no family history of fibroadenomas or cancer of the breast, no hypertension, no diabetes mellitus, or autoimmune disease).

### Blood samples

Five ml of Whole blood was collected from each subject and divided into 2 portions. Two ml in EDTA collection tubes for DNA extraction. The other 3 ml was centrifuged in a plain tube to give serum that was used for RNA extraction and real-time PCR.

### RNA extraction& cDNA synthesis

The TRIzol reagent was used to extract serum's total RNA, including lncRNA (Zymo Research, Irvine, CA). RNA was measured using NanoDrop2000 (Thermo Fischer Scientific, Waltham, MA). The whole RNA samples were stored at − 80 °C before use. Reverse transcription was carried out on the RNA using SensiFAST cDNA Synthesis Kit [Bioline, Memphis, TN] in a final volume of 20 μl reactions.

### Real time PCR

According to the manufacturer’s instructions, the Hera plus SYBR Green qPCR kit [Willow fort, Birmingham, UK] was used to measure the expression levels of HULC and MTMR3 using GAPDH as an internal control.

Fold change was calculated using the comparative threshold cycle [2^−ΔΔCt^] for relative quantification normalized to an endogenous control [14].

The primer sequences were as follows:

**Table:** 

Gene	Forward	Reverse
MTMR3	5′-AGCAGAGTGGGCTCAGTGTT-3′	5′-ACTGTCCACGTTTGGT-CCTC-3′
HULC	5′-TCATGATGGAATTGGAGCCTT-3′	5′-CTCTTCCTGGCTTGCAGATTG-3′
GAPDH	5′-CCCTTCATTGACCTCAACTA-3′	5′-TGGAAGATGGTGATGGGATT-3′

The following conditions were used for doing real-time PCR on a 20 ml reaction mixture using Applied Biosystems’ 7500 Real-Time PCR Systems: 40 cycles at 95 °C for 15 s and 60 °C for 60 s were performed after 95 °C for 10 min.

### DNA extraction

DNA was extracted from whole blood using Qia-amplification DNA extraction kit (Qiagen, USA) according to manufacturer’s instructions. NanoDrop2000 (Thermo scientific, USA) was used to assess the yield.

### Genotyping of SNP in MEG3 rs7158663

Genotyping was done using real-time polymerase chain reaction with TaqMan allelic discrimination assay (Applied Biosystems, USA). Predesigned primer/probe sets for rs7763881 and rs12537 were used.

### PCR amplification protocol

The 25 μl total volume used for DNA amplification contained 12.5 μl Taqman master mix, 1.25 primer/probe, 1 μl (100 μg)of DNA, and 10.25 μl water. Real-time PCR was performed using the Rotor gene Q Real Time PCR System (Qiagen, Valencia, CA, USA) under the following circumstances: 10 min at 95 °C, followed by 45 cycles of 15 s each at 92 °C then 60 °C for 90 s. At the end of each cycle and at the endpoint, fluorescence was measured.

### Statistical analysis

For Single Nucleotide Polymorphism (SNP) analysis, data were entered and analyzed using the online SNPStats software https://www.snpstats.net/start.htm. This involves allele frequencies, genotype frequencies, Hardy–Weinberg equilibrium (HWE), and testing for multiple inheritance models: co-dominant, dominant, recessive, over-dominant and additive models. Data were also entered and analyzed using IBM-SPSS software (IBM Corp. Released 2019. IBM SPSS Statistics for Windows, Version 26.0. Armonk, NY: IBM Corp).Qualitative data were expressed as N (%) and compared by Chi-square test. Quantitative data were initially tested for normality using Shapiro–Wilk’s test and presence of significant outliers was tested for by inspecting boxplots. Non-normally distributed data were expressed as median (Q1–Q3) and compared by Kruskal–Wallis H-test. Spearman’s correlation was used to assess the direction and strength of association between two quantitative variables. ROC curves were used to find a cutoff value that discriminate those with and without a certain condition. Binary logistic regression was used to ascertain the effects of predictor variables on the likelihood that participants will exhibit a certain condition. For any of the used tests, results were considered as statistically significant if p value ≤ 0.050.Appropriate charts were used to graphically present the results whenever needed.

## Result

This study involved 245 female participants, divided into 4 groups: group A (non-metastatic breast cancer) = 85 females; group B (metastatic cancer) = 40 females; group C (fibroadenoma) = 40 females; and group D (age-matched healthy control) = 80 females. Clinical and demographic data are shown in Table 1.

**Table 1:** Clinical and demographic data (n = 245)

Characteristic	N	%
Postmenopausal	113	46.1
Diabetes	17	6.9
Hypertension	21	8.6
Positive family history	62	25.3
Hormonal therapy	4	1.6

Characteristic	Median	Q1–Q3
Age (years)	49	43.5–57.5
Body weight (kg)	69	62–77
Parity among the married (n = 225)	2	2–3
Fasting blood glucose (mg/dl)	104	100.5–112
AST (IU/L)	26.5	19–41.7
ALT (IU/L)	18.5	15–37.5
Alkaline phosphatase	96.5	73–143.2
Serum total bilirubin (mg/dl)	0.5	0.4–0.6

### HULC rs7763881, MTMR3 rs12537 SNPs analysis

Our results show a statistically low but significant association between MTMR3 alleles and breast cancer. The “T” allele is a risk allele for breast cancer; participants with the “T” allele of the MTMR3 gene are twice as likely as those with the “C” allele to develop breast cancer. The HULC alleles, on the other hand, did not show a statistically significant association with breast cancer (Table 2).

**Table 2:** Allele frequencies and association

Allele/Genotype	N (%)			Chi-square test			Binary logistic regression		
	Case (%)	Control (%)	Total (%)	χ^2^	φ	P-value	COR	95% CI	P-value
MTMR3				11.999	0.171	**0.001**			**0.001**
‘C’	100 (40)	92 (57.5)	192 (46.8)				r(1)	r(1)	
‘T’	150 (60)	68 (42.5)	218 (53.2)				2.03	1.36–3.04	
C/C	29 (23.2) a	23 (28.7)^a^	52 (25.4)	20.426	0.316	** < 0.001**	r(1)	r(1)	
C/T	42 (33.6)^a^	46 (57.5)^b^	88 (42.9)				0.72	0.36–1.442	0.358
T/T	54 (43.2) a	11 (13.8)^b^	65 (31.7)				3.89	1.67–9.095	**0.002**
HULC				2.374	0.076	0.123			0.124
‘A’	118 (47.2)	88 (55)	206 (50.2)				r(1)	r(1)	
‘C’	132 (52.8)	72 (45)	204 (49.8)				1.37	0.92–2.04	
A/A	26 (20.8)	21 (26.3)	47 (22.9)	3.069	0.122	0.216	r(1)	r(1)	
A/C	66 (52.8)	46 (57.5)	112 (54.6)				1.16	0.58–2.304	0.674
C/C	33 (26.4)	13 (16.3)	46 (22.4)				2.05	0.87–4.854	0.102

Bold indicate statistically significant

The MTMR3 genotypes and breast cancer had a statistically significant but moderate association. The “C/T” genotype is more prevalent in control than in case, whereas the “T/T” genotype is more prevalent in case than in control (risk genotype). Participants with ‘T/T’ genotype of the MTMR3 gene have 3.9-times higher odds of developing breast cancer than those with ‘C/C’ genotype. The HULC genotypes, on the other hand, did not show a statistically significant correlation with breast cancer (Table 2). The observed genotype frequencies for both genes in control group are consistent with Hardy–Weinberg equilibrium (P = 0.114 for MTMR3 and 0.148 for HULC).

We investigated the best inheritance model of the MTMR3 and HULC genes adjusted for age and discovered that the T/T genotype of the MTMR3 gene is statistically significantly higher in cases compared to controls compared to C/C–C/T genotypes with 4.9-times higher odds to exhibit the disease. There is no statistically significant inheritance model for the HULC gene. However, the C/C genotype of the HULC gene is more prevalent in cases compared to controls, with 1.9 fold higher odds of developing the disease (Table 3).

**Table 3:** Best inheritance model of MTMR3 and HULC genes (adjusted by age)

Gene	Model	Genotypes	Case (%)	Control (%)	AOR (95% CI)	p-value	AIC	BIC
MTMR3	Recessive	C/C-C/T	71 (56.8)	69 (86.2)	r(1)	** < 0.001**	258.1	268
		T/T	54 (43.2)	11 (13.8)	4.9 (2.3–10.1)			
HULC	Recessive	A/A-A/C	92 (73.6)	67 (83.8)	r(1)	0.072	276.3	286.2
		C/C	33 (26.4)	13 (16.2)	1.9 (0.93–3.91			

Bold indicate statistically significant

*AOR* adjusted odds ratio, *CI* confidence interval, *AIC* Akaiki information criterion, *BIC* Bayesian information criterion, *r(1)* reference category. Test of significance is binary logistic regression

### Serum levels of MTMR3 mRNA and HULC mRNA in BC patients

When the four study groups’ serum levels of MTMR3 mRNA and HULC mRNA were examined, Table 4 revealed a statistically significant difference in the expression levels of the two genes. Pairwise comparisons revealed that MTMR3 gene expression was higher in both group A and group B compared to both group C and group D (P < 0.001 for all pairwise comparisons), but not between group A and group B (P = 1.000). It was also higher in-group C when compared to group D (P < 0.001).HULC gene expression was higher in-group A > group B > group C > group D (P-value = 0.003 for group C vs. group D, = 0.028 for group A compared to group B, P < 0.001 for all other pairwise comparisons).

**Table 4:** Gene expression in the three group

Gene	Group A	Group B	Group C	Group D	H [3]	P-value
	N = 85	N = 40	N = 40	N = 80		
MTMR3	17.562 (8.398–50.816)	23.615 (12.423–41.625)	1.400 (1.203–1.978)	1 (1–1)	168.391	** < 0.001**
	A	A	B	C		
HULC	4.075 (1.955–9.558)	9.295 (3.298–13.808)	1.200 (1.043–1.973)	1 (1–1)	147.357	** < 0.001**
	A	B	C	D		

Bold indicate statistically significant

Data is median (Q1–Q3). Test of significance is Kruskal–Wallis H-test. Pairwise comparisons with Bonferroni correction for multiple tests is presented as capital letters (similar letters = insignificant difference, and different letters = significant difference)

### The effect of HULC rs7763881 and MTMR3 rs12537 on serum gene expression

Our results (Table 5) show a statistically significant difference in MTMR3 and HULC gene expression between the three genotypes for each gene. Pairwise comparisons revealed that MTMR3 gene expression was higher in the T/T genotype compared to both the C/C and C/T genotypes (P < 0.001 for all pairwise comparisons), but not between the C/C and C/T genotypes (P = 1.000). In addition, pairwise comparisons revealed that HULC gene expression was lower in the A/C genotype compared to both the A/A and C/C genotypes (P = 0.036 and < 0.001, respectively), but not between the A/A and C/C genotypes (P = 0.270).

**Table 5:** Gene expression in the three genotypes

Gene	Genotypes			H [3]	P-value
MTMR3	C/C	C/T	T/T	44.675	< 0.001
	1.55 (1.0–12.24)	1.17 (1.0–6.15)	22 (11.0–62.7)		
	A	A	B		
HULC	A/A	A/C	C/C	21.559	< 0.001
	2.0 (1.0–8.8)	1.07 (1.0–2.8)	5.12 (1.44–10.24)		
	A	B	A		

Data is median (Q1–Q3). Test of significance is Kruskal–Wallis H-test. Pairwise comparisons with Bonferroni correction for multiple tests is presented as capital letters (similar letters = insignificant difference, and different letters = significant difference)

## Diagnostic performance of serum MTMR3 and HULC

**Fig. 1:**
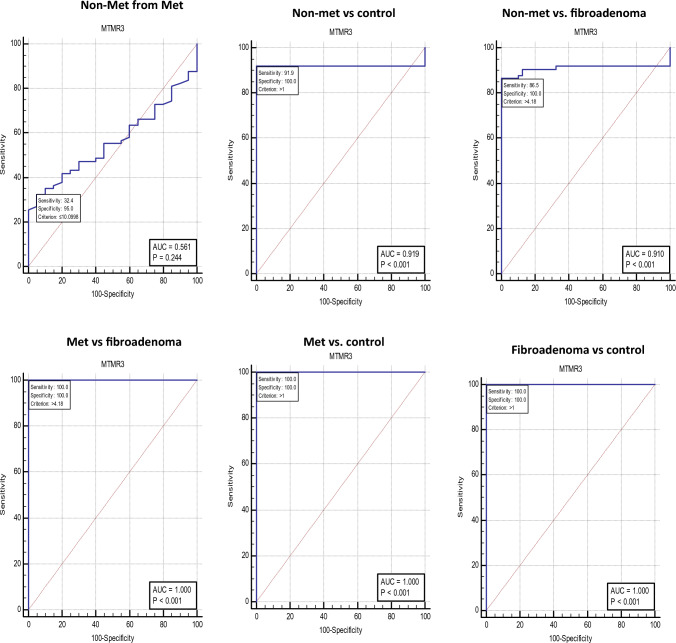
Diagnostic performance of serum MTMR3. ROC curve analysis of serum MTMR3 to discriminate studied groups, BC non metastatic (n = 85), BC metastatic (n = 40), fibroadenoma (n = 40), and healthy controls (n = 80)

HULC (Fig. 1) is a statistically significant discriminator of all 4 groups: non-metastatic from metastatic (cutoff ≤ 2.356), non-metastatic from fibroadenoma (cutoff ≤ 2.357), non-metastatic from control (cutoff > 1.0), metastatic from fibroadenoma (cutoff > 2.88), metastatic from control (cutoff > 1.0), and fibroadenoma from control (cutoff > 1.0).

**Fig. 2:**
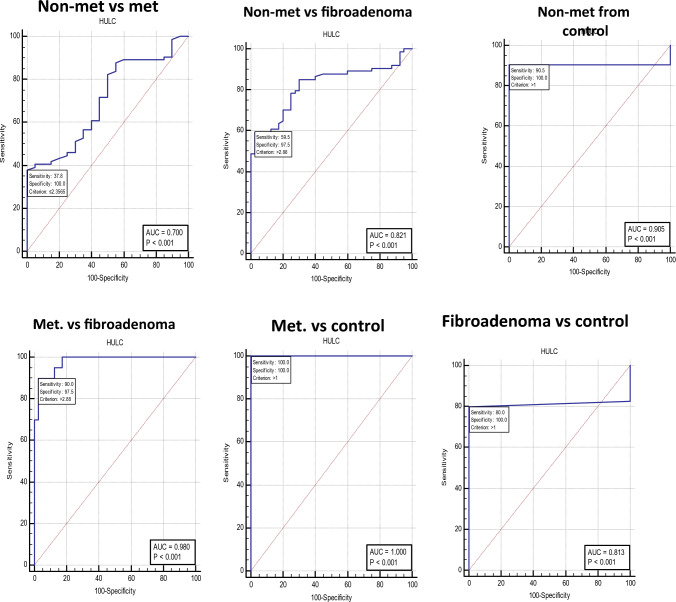
Diagnostic performance of serum HULC. ROC curve analysis of serum HULC to discriminate studied groups, BC non-metastatic (n = 85), BC metastatic (n = 40), fibroadenoma (n = 40), and healthy controls (n = 80)

While MTMR3 (Fig. 2) is a statistically significant discriminator of all four groups (except non-metastatic from metastatic): non-metastatic from fibroadenoma (cutoff > 4.18), non-metastatic from control (cutoff > 1.0), metastatic from fibroadenoma (cutoff > 4.18), metastatic from control (cutoff > 1.0), and fibroadenoma from control (cutoff > 1.0).

### Correlation of HULC rs7763881, MTMR3 rs12537 genotypes, serum MTMR3, and HULC levels with clinicopathological data

Our findings revealed a highly positive correlation between MTMR3 and HULC that was statistically significant. The medium-strength positive correlation between MTMR3 and ALT as well as between HULC and alkaline phosphatase were both statistically significant (Table 6).

**Table 6:** Correlations of the two gene expressions

Characteristic	MTMR3		HULC	
	r_s_	p-value	r_s_	P-value
MTMR3	–	–	0.757	** < 0.001**
HULC	0.757	** < 0.001**	–	–
Age (years)	0.109	0.098	0.096	0.143
Body weight (kg)	− 0.220	0.059	− 0.079	0.505
Fasting blood glucose (mg/dl)	− 0.116	0.475	0.041	0.799
AST (IU/L)	− 0.273	0.088	0.221	0.170
ALT (IU/L)	− 0.350	**0.027**	0.069	0.670
Alkaline phosphatase	0.239	0.138	0.361	**0.022**
Serum total bilirubin (mg/dl)	− 0.126	0.437	− 0.003	0.985
Tumor grade	0.259	0.107	0.020	0.903

Bold indicate statistically significant

*r*_*s*_ Spearman’s correlation coefficient

## Discussion

Breast cancer is the most popular cancer in women worldwide. Regarding deaths from cancer, breast cancer is responsible for 15% of deaths from cancers [1].Many publications have investigated the important roles of many genes in the susceptibility to breast carcinoma [15]. MTMR3 was reported to contribute to immunological diseases such as RA and SLE, as well as gastric and breast cancer [16]. While HULC is one of the LncRNAs that are highly expressed in liver cancer cells, it plays a vital role in various cellular processes, including cell proliferation and apoptosis [7]. Based on this, we sought to examine the relationship between the HULC rs7763881 and MTMR3 rs12537 SNPs, clinicopathological data, their correlation with the expression of HULC and MTMR3 in serum, and their association with the susceptibility to BC and fibroadenoma. Additionally, we looked at the serum levels of HULC and MTMR3, their connection with clinicopathological data, and their potential as non-invasive biomarkers of BC in fibroadenoma and BC patients.

In our analysis, homozygocity for the T allele at MTMR3 rs12537 was associated with an elevated risk for breast cancer, whereas the CC genotype had no statistically significant effect. According to these findings, this SNP might be a propensity SNP for BC development. Similar findings were found to MTMR3 T allele genotype rs12537, which showed a high risk and poor prognosis of gastric cancer [17].

We demonstrated that MTMR3 gene expression was higher in both metastatic and non-metastatic groups and was also increased in the T/T genotype compared to both C/C and C/T genotypes. MTMR3 was positively correlated with ALT. Similar to this, MTMR3 expression was considerably higher in breast cancer tissues compared to normal tissues [17]. High MTMR3 expression levels may be detrimental to patients with breast cancer in terms of overall survival and relapse-free survival. If MTMR3 is overexpressed, autophagy is reduced [8]. Instead, MTMR3 expression was lower in breast cancer tissues than in normal tissues, but it was heavily methylated, suggesting that the rs12537 mutation in patients with estrogen receptor-positive breast cancer may control MTMR3 methylation [18].

Our findings revealed that the HULC gene was expressed more strongly in BC. Both a statistically significant inheritance model and an association between the HULC rs7763881 allele and breast cancer were absent. However, the C/C genotype of the HULC gene is more common in cases than in controls, as opposed to the A/A-A/C genotype. Additionally, HULC gene expression was lower in the A/C genotype compared to both the A/A and C/C genotypes.

Similar to this, HULC expression was higher in breast cancer tissues compared to healthy, normal breast tissues [7]. In many malignancies, such as ovarian cancer and prostate cancer, HULC behave as oncogenes that may speed up the disease progression [6]. Also, lncRNA HULC shows higher levels in hepatocellular cancer and increases its progression [19]. Additionally, downregulation of HULC leads to p53 stimulation, increased p21 expression, and finally apoptosis [20].

It was reported that HULC apparently inhibited apoptosis through an increase in Bcl-2 expression levels. HULC depletion leads to a decrease in Bcl-2 and an increase in the Bax/Bcl-2 expression ratio in breast cancer cell lines. An increased Bax/Bcl-2 ratio leads to upregulation of caspase-3 and increases apoptosis [21]. Furthermore, Wang et al. have shown that HULC expression is elevated in HCC patients and that HULC significantly contributes to tumour development by suppressing miR-372 [22].

The tumour suppressor genes P18 and miR-372 may both be downregulated by the lncRNA HULC, increasing the proliferation of HCC cells. It was also shown that HULC regulates IGF1R expression, which in turn affects the PI3K/AKT pathway, which is a downstream effect. Breast cancer development and metastasis are promoted by the activated HULC-IGF1R pathway [23].

The observed positive correlation between MTMR3 and HULC is consistent with their effect on autophagy. MTMR3 decreased pattern recognition receptor (PRR)-induced PI3P and autophagy levels by affecting ATG5 [24]. HULC expression levels were increased in ovarian cancer by decreasing ATG7 to decrease autophagy [25].

In this study, we revealed a positive correlation between HULC and alkaline phosphatase, confirming that HULC can be used as a marker for diagnosing breast cancer [26]. Correlated alkaline phosphatase with metastasis, and thus it is considered a useful marker of metastasis.

The ROC curve was created to examine MTMR3’s and HULC’s diagnostic abilities, HULC was a statistically significant discriminator of all 4 groups: non-metastatic from metastatic (cutoff ≤ 2.356), non-metastatic from fibroadenoma (cutoff ≤ 2.357), non-metastatic from control (cutoff > 1.0), metastatic from fibroadenoma (cutoff > 2.88), metastatic from control (cutoff > 1.0), and fibroadenoma from control (cutoff > 1.0). MTMR3 was a statistically significant discriminator of all four groups (except non-metastatic from metastatic): non-metastatic from fibroadenoma (cutoff > 4.18), non-metastatic from control (cutoff > 1.0), metastatic from fibroadenoma (cutoff > 1.0), and fibroadenoma from control (cutoff > 1.0). Due to their high ability to distinguish between cases and controls, both genes demonstrated a good predictive value in BC, proposing serum MTMR3 and HULC as viable non-invasive biomarkers for BC diagnosis.

### Supplementary Information

Below is the link to the electronic supplementary material.

Supplementary file1 (DOCX 14 KB)

## Data Availability

The datasets generated during and/or analysed during the current study are available from the corresponding author on reasonable request.
