# Supplementary material for: Breast cancer risk is associated with the HULC rs7763881, MTMR3 rs12537 polymorphisms, and serum levels of HULC and MTMR3 in Egyptian patients
Source: Mol Biol Rep. 2023 Nov 1;50(12):10073–81. doi: 10.1007/s11033-023-08897-1 (PMC10676311; doi:10.1007/s11033-023-08897-1)
Supplement: Supplementary file 1 — Supplementary file1 (DOCX 14 KB) [file 11033_2023_8897_MOESM1_ESM.docx]

**Value of MTMR3 and HULC gene expressions in discriminating the 4 groups**

| Pair to be discriminated | Gene | Cutoff value | AUC (95% CI) | p-value | Sensitivity | Specificity |
| --- | --- | --- | --- | --- | --- | --- |
| Group A from group B | MTMR3 | ≤10.1 | 0.561 (0.465-0.654) | 0.244 | 32.4% | 95% |
|  | HULC | ≤2.357 | 0.700 (0.607-0.783) | **<0.001** | 37.8% | 100% |
| Group A from group C | MTMR3 | >4.18 | 0.910 (0.842-0.955) | **<0.001** | 86.5% | 100% |
|  | HULC | >2.88 | 0.821 (0.738-0.886) | **<0.001** | 0.59.5 | 97.5% |
| Group A from group D | MTMR3 | >1.0 | 0.919 (0.864-0.957) | **<0.001** | 91.9% | 100% |
|  | HULC | >1.0 | 0.905 (0.848-0.947 | **<0.001** | 90.5% | 100% |
| Group B from group C | MTMR3 | >4.18 | 1.000 (0.955-1.000) | **<0.001** | 100% | 100% |
|  | HULC | >2.88 | 0.980 (0.920-0.998) | **<0.001** | 90% | 97.5% |
| Group B from group D | MTMR3 | >1.0 | 1.000 (0.970-1.000) | **<0.001** | 100% | 100% |
|  | HULC | >1.0 | 1.000 (0.970-1.000) | **<0.001** | 100% | 100% |
| Group C from group D | MTMR3 | >1.0 | 1.000 (0.970-1.000) | **<0.001** | 100% | 100% |
|  | HULC | >1.0 | 0.813 (0.731-0.878) | **<0.001** | 80% | 100% |

Notes: AUC = area under the ROC curve. CI = confidence interval.
